# Un vasospasme fatal compliquant une hémorragie méningée grave

**DOI:** 10.11604/pamj.2015.20.346.5863

**Published:** 2015-04-10

**Authors:** Soumaya Touzani, Mohammed Khatouf

**Affiliations:** 1 Service de Réanimation Polyvalente A1, CHU Hassan II de Fès, Maroc

**Keywords:** Hémorragie méningée grave, vasospasme, anévrysme, subarachnoid hemorrhage, vasospasm, aneurism

## Image en medicine

**Figure 1:**
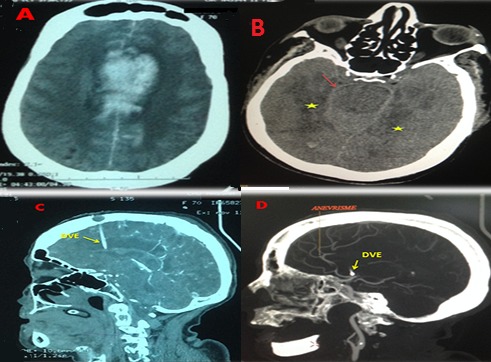
(A) hémorragie méningée avec inondation ventriculaire bilatérale au scanner initial; (B) persistance de l'hémorragie méningée (flèche rouge) + apparition de plages ischémiques fronto-temporo-pariétales bilatérales (étoiles jaunes) sur l'angioscanner; (C) hypodensités en rapport avec le vasospasme D) anévrysme de l'artère cérébrale antérieure droite. DVE (dérivation ventriculaire externe)

Il s'agit d'une patiente de 70 ans, diabétique sous insulinothérapie, admise aux urgences en troubles de conscience. Le scanner cérébral à son admission a montré une hémorragie méningée grade IV de WFNS grade 4 de Fisher avec inondation ventriculaire et hydrocéphalie (A). La patiente a bénéficié d'une dérivation ventriculaire externe. L'angioscanner cérébral (B, C, D) réalisé à H24 a objectivé une persistance de l'hémorragie méningée avec inondation ventriculaire (B), un accident vasculaire ischémique dû au vasospasme dans les territoires des artères cérébrales antérieures et postérieures en bilatéral (B, C) et un petit anévrysme de l'artère cérébrale antérieure de 3 millimètres (D). Les vélocités moyennes au doppler transcrânien étaient élevées (210 cm/s). La prise en charge thérapeutique associait nimodipine en perfusion continue, hyperventilation et « triple-H thérapie». L’évolution était fatale par l'installation rapide d'une défaillance multiviscérale. Le vasospasme qui suit l'hémorragie méningée anévrismale est une pathologie complexe à l’étiologie multifactorielle. Défini comme une réduction de la lumière d'une artère conductrice dans l'espace sous-arachnoïdien associée à des troubles de la microcirculation, il survient classiquement dès le troisième jour et jusqu’à la quatrième semaine après l'hémorragie méningée. L'observation d'un vasospasme ultraprécoce a valeur de resaignement et serait prédictif d'une lourde morbi-mortalité. Il peut conduire à des « déficits neurologiques ischémiques retardés » et parfois au décès. Le traitement préventif repose sur la nimodipine et une fois le vasospasme avéré et l'anévrysme sécurisé, la « triple-H thérapie » (hypervolémie, hypertension artérielle et hémodilution) est préconisée. Figure 1

